# Gesundheit und Klimawandel – welche Potenziale haben versorgungsnahe Daten?

**DOI:** 10.1007/s00103-023-03828-8

**Published:** 2024-01-19

**Authors:** Christian Günster, Caroline Schmuker

**Affiliations:** Wissenschaftliches Institut der AOK (WIdO), Rosenthaler Straße 31, 10178 Berlin, Deutschland

**Keywords:** Public Health, Versorgungsforschung, Routinedaten, Vulnerabilität, Morbidität, Public health, Health services research, Routine data, Vulnerable population, Morbidity

## Abstract

Dieser Beitrag geht der Frage nach, welche Auswirkungen der Klimawandel auf die Gesundheit haben kann und inwieweit versorgungsnahe Daten zur Forschung in diesem Themenfeld beitragen können. Der Klimawandel verändert die Umwelt- und Lebensbedingungen der Menschheit, er ist damit auch zu einem relevanten Gesundheitsproblem geworden. Die Zunahme von Extremwetterereignissen, Veränderungen bei der UV- und Luftschadstoffbelastung sowie die klimaassoziierte Verbreitung von Allergenen oder neuartigen Erregern verändern das Spektrum an Erkrankungen und den medizinischen Versorgungsbedarf in der Bevölkerung erheblich. Allerdings liegen bislang nur wenige Erkenntnisse zu den Folgen für das Gesundheitssystem und zu besonders betroffenen Bevölkerungsgruppen in Deutschland vor. Versorgungsnahe Daten (Primärdaten, Registerdaten, Sekundärdaten) in Verbindung mit Umweltexpositionsdaten und modulierenden Daten (z. B. sozioökonomische Daten) haben das Potenzial, die Forschung zu den gesundheitlichen Folgen des Klimawandels erheblich voranzubringen. Dieser Beitrag benennt die Veränderungen der Umwelt- und Lebensbedingungen sowie der damit verbundenen gesundheitlichen Risiken. Er beschreibt die Datengrundlagen, die grundsätzlich zur Analyse gesundheitlicher Auswirkungen des Klimawandels zur Verfügung stehen. An einem konkreten Beispiel wird aufgezeigt, wie die Zusammenführung von individuellen Gesundheitsdaten (hier GKV-Abrechnungsdaten), Umweltexpositionsdaten und modulierenden Daten gelingen kann. Der Beitrag bietet abschließend eine umfassende Übersicht über offene Forschungsfragen, die mit versorgungsnahen Daten beantwortet werden können.

## Abstract (en)

This article addresses the question of how climate change may affect health and to what extent real-world data can contribute to research in this topic area. Climate change is altering the environmental and living conditions of humankind, and has thus also become a relevant health problem. The increase in extreme weather events, changes in exposure to UV and air pollution, and the climate-associated spread of allergens or novel pathogens are significantly changing the spectrum of diseases and the need for medical care in the population. However, in Germany, only few findings on the consequences for the healthcare system and on particularly affected population groups exist so far. Real-world data (primary data, register data, and administrative data) in combination with environmental exposure data and other relevant data (e.g., socio-economic data) have the potential to significantly advance research on the health consequences of climate change. This paper identifies changes in environmental and living conditions and associated health risks. It describes the databases that are generally available for analysing health effects of climate change. A concrete example is used to show how individual health data (in this case claims data of the statutory health insurance), environmental exposure data and other data can be successfully combined. Finally, the article offers a comprehensive overview of open research questions that can be answered with real-world data.

## Einleitung

Der Klimawandel hat Auswirkungen, die weit über Umweltaspekte hinausgehen. Er hat sich zu einem relevanten globalen Gesundheitsproblem entwickelt. Die direkten Auswirkungen des anthropogenen Klimawandels auf die Umwelt- und Lebensbedingungen sind vielfältig. Im Vordergrund steht die globale Erderwärmung. Der Weltklimarat (Intergovernmental Panel on Climate Change – IPCC) betrachtet in seinem 6. Sachstandsbericht die Erderwärmung in verschiedenen Szenarien (Shared Socioeconomic Pathways – SSP) der Entwicklung in Bezug auf Treibhausgasemissionen und Klimaschutzmaßnahmen [1]. In allen betrachteten Emissionsszenarien wird die globale Oberflächentemperatur bis mindestens Mitte des 21. Jahrhunderts weiter ansteigen. Die Bandbreite der Erwärmung (Durchschnitt der Jahre 2081–2100 im Vergleich zur vorindustriellen Zeit) würde sich im Szenario mit sehr niedrigen Treibhausgasemissionen (SSP1‑1.9) zwischen 1,0 und 1,8 °C bewegen und im Szenario mit sehr hohen Treibhausgasemissionen (SSP5‑8.5) zwischen 3,3 und 5,7 °C. Selbst im Szenario mit mittleren Treibhausgasemissionen (SSP2‑4.5) ergäbe sich eine Erwärmung um 2,1 °C bis 3,5 °C. Ohne radikale Reduktion der Treibhausgasemissionen würden demnach die Schwellenwerte von 1,5 °C und 2 °C im Laufe des Jahrhunderts überschritten werden.

Direkte Auswirkungen der globalen Erderwärmung zeigen sich mittelbar in Form der Zunahme von Extremereignissen wie Hitzewellen, Trockenheit und Dürren, Waldbränden, Stürmen und Starkregen [2]. Mit diesen direkten Folgen sind zum Teil tiefgreifende sekundäre Veränderungen der uns umgebenden natürlichen Systeme verbunden [3]. Zum Beispiel trägt der Klimawandel dazu bei, Vegetationsperioden zu verschieben und zu verlängern und das Auftreten neuer allergener Pflanzen in Europa zu befördern [4]. Konkret ist zu beobachten, dass sich die Pollenflugsaison ausdehnt und sich Pollenarten in der Atmosphäre verbreiten, die in Deutschland bisher nicht vorkamen.

Die Veränderungen des Klimas und der Ökosysteme haben Auswirkungen auf die menschliche Gesundheit in Form von hitzeassoziierten Erkrankungen, Infektionserkrankungen, allergischen Reaktionen, Erkrankungen im Zusammenhang mit Luftschadstoffen und ultravioletter(UV-) Strahlung sowie psychischen Belastungen [5–12]. Bezüglich der Auswirkungen des Klimawandels auf die Gesundheitsversorgung und das Gesundheitssystem besteht erheblicher Forschungsbedarf [3, 13]. Das gilt zudem für die Untersuchung der Wirksamkeit von Maßnahmen zur Prävention gesundheitlicher Folgen des Klimawandels und der Klimaanpassung. Die Versorgungsforschung ist durch ihren Rückgriff auf versorgungsnahe Daten besonders geeignet, Art, Umfang und Struktur der Klimawandelfolgen auf die Gesundheitsversorgung zu untersuchen.

Zielsetzung dieses Beitrags ist es, die Themenbereiche der Auswirkungen des Klimawandels auf die Gesundheit und das Gesundheitssystem zu benennen und aufzuzeigen, welche Relevanz versorgungsnahe Daten für die Untersuchung der Klimawandeleffekte in Deutschland haben. Dazu werden zunächst ausgehend von den Veränderungen der Umwelt- und Lebensbedingungen die potenziellen gesundheitlichen Folgen der Klimaveränderungen beschrieben. Zur Verfügung stehende Datengrundlagen werden erläutert und ihre Verwendung an einem Beispiel vorgestellt, wobei auf Herausforderungen und Potenziale eingegangen wird.

## Auswirkungen des Klimawandels auf die Umwelt- und Lebensbedingungen

Folgen des Klimawandels auf die Umwelt und Lebensbedingungen in Deutschland zeigen sich sowohl im vermehrten Auftreten von Extremereignissen als auch in langsam fortschreitenden Veränderungen der uns umgebenden Systeme (siehe Infobox 1).

Zunächst sind Hitzewellen zu nennen. Sie lassen sich hinsichtlich ihrer Dauer und Intensität charakterisieren. Während für die Jahre 1994, 2003, 2006, 2013 und 2015 für Deutschland lediglich einzelne markante Hitzesommer berichtet wurden [14, 15], sind seit 2018 jährlich Hitzewellen mit mindestens drei aufeinander folgenden Tagen mit einer Tageshöchsttemperatur von über 30 °C (sog. Hitzetage) zu verzeichnen. Im Höhepunkt der Hitzewelle des Juli 2019 wurden an 25 Messstationen des Deutschen Wetterdienstes sogar Temperaturen über 40 °C gemessen [16]. Die Anzahl der Hitzewellen pro Jahr sowie ihre jeweilige Dauer und mittlere Lufttemperatur werden weiter zunehmen [12, 17, 18]. Dabei sind die Hitzetage in Deutschland regional sehr unterschiedlich verteilt. Der Oberrheingraben, das Rhein-Main-Gebiet und die Lausitz sind besonders betroffen [12]. Unter den Bundesländern hatte Berlin in den Jahren 2011 bis 2020 mit durchschnittlich 15,7 Hitzetagen pro Jahr die meisten Hitzetage zu verzeichnen, während Schleswig-Holstein mit 3,7 Tagen die geringste Belastung aufwies [19].

Von Dürren ist der globale Norden weltweit betrachtet vergleichsweise wenig betroffen. Phasen anhaltender Trockenheit sind jedoch auch in Zentraleuropa und Deutschland verstärkt zu beobachten [2]. Trockenperioden gehen oft mit einer Verschlechterung der Luftqualität durch Zunahme von Luftschadstoffen und Staubpartikeln einher [20]. Lange und großräumige Trockenphasen führen zu niedrigen Pegelständen in Flüssen, Seen und Talsperren mit potenziellen Auswirkungen auf die Wasserqualität durch erhöhte Schadstoff- und Keimkonzentrationen sowie in Verbindung mit erhöhten Wassertemperaturen und Einschwemmungen auf das Risiko wasserbürtiger Infektionen [10].

Eine weitere Folge von steigenden Temperaturen und Dürren ist die Erhöhung der Waldbrandgefahr. Am häufigsten sind Tage mit hoher bis sehr hoher Waldbrandgefahr in den östlichen Bundesländern sowie im Rhein-Main-Gebiet [21]. Im Osten Deutschlands und am Oberrhein sind im pessimistischen Klimaszenario bis zur Mitte des Jahrhunderts im Mittel mehr als 40 Tage mit hoher oder sehr hoher Waldbrandgefahr zu erwarten. Die Waldbrandgefahr muss jedoch vom konkreten Eintreten eines Waldbrandes unterschieden werden, da die Hälfte der Waldbrände in Deutschland fahrlässig oder vorsätzlich verursacht wird.

Schließlich ist in Deutschland als Folge der Erwärmung ein Trend zu wärmeren Wintern und der Abnahme von Schnee- und Eistagen festzustellen. Bei fortschreitender Erderwärmung werden bis Ende des Jahrhunderts weite Teile Deutschlands vollständig schneefrei bleiben [2]. Extremniederschläge haben in Deutschland dagegen überproportional zugenommen; eine weitere Zunahme ihrer Häufigkeit und Heftigkeit wird erwartet [2]. Ein Starkregenereignis, wie es 2021 in Rheinland-Pfalz und Nordrhein-Westfalen aufgetreten ist, ist durch den anthropogen verstärkten Klimawandel wahrscheinlicher geworden [22].

Die Veränderung der Belastung durch UV-Strahlen wird durch eine Vielzahl von Faktoren beeinflusst. Der wichtigste Einflussfaktor ist – unabhängig vom Klimawandel – die stratosphärische Ozonkonzentration. Die vollständige Regeneration des stratosphärischen Ozons nach Reglementierung des Eintrags von ozonzerstörenden Substanzen seit der 1990er-Jahre wird für Mitte des 21. Jahrhunderts erwartet. Unabhängig davon scheinen jedoch in mittleren Breiten Veränderungen der Bewölkung wesentlich die bisherigen Änderungen der bodennahen UV-Strahlung hervorgerufen zu haben [23].

Bereits angesprochen wurde die erhöhte Allergenexposition in Folge des Klimawandels. Ursachen hierfür sind unter anderem die höhere Pollenproduktion und der frühere Beginn der Pollensaison an Standorten mit erhöhten Temperaturen sowie generell in wärmeren Jahren. Begünstigend wirkt sich dabei auch der Anstieg der frostfreien Tage pro Jahr aus. Als Folge der sich verändernden Klimabedingungen ist die Ausbreitung neuer, invasiver Pflanzenarten in Deutschland festzustellen wie zum Beispiel der Ambrosiapflanze, deren Pollen besonders stark allergen sind [4]. Einen weiteren Faktor für zunehmende Allergien stellt das vermehrte Schimmelpilz- und Bakterienwachstum nach Starkregen und Überschwemmungen dar [7].

Höhere Temperaturen und veränderte Niederschlagsmuster beeinflussen weiterhin das Auftreten und die Verbreitung von Vektor- und Nagetier-assoziierten Erregern [6]. Ein Beispiel ist die Ausweitung der Risikogebiete für Infektionen mit dem Frühsommer-Meningoenzephalitis(FSME)-Virus durch den Kontakt mit Zecken. Von 129 betroffenen Stadt- und Landkreisen im Jahr 2007 ist deren Anzahl durch eine Ausweitung überwiegend nach Norden auf 175 im Jahr 2022 angewachsen [24]. Zecken profitieren von wärmeren Temperaturen insbesondere im Winter und Frühling. Die veränderten Klimabedingungen begünstigen zudem die Etablierung neozoischer, vektor-kompetenter Stechmückenarten in Deutschland und damit die Verbreitung von Viren, die von diesen oder bereits heimischen Stechmückenarten übertragen werden. So ist im Jahr 2018 erstmals das West-Nil-Virus in Deutschland nachgewiesen worden.

Im Zuge des Klimawandels und der damit verbundenen erhöhten Luft- und Wassertemperaturen, zunehmenden Niederschlägen, aber auch Trockenperioden können die Verbreitung von Toxinen sowie die Verbreitung, Vermehrung und das Überleben von lebensmittelassoziierten Erregern in tierischen und pflanzlichen Lebensmitteln begünstigt werden [9]. Beispielsweise kann eine erhöhte Umgebungstemperatur die Entwicklung von Salmonellen innerhalb der Lebensmittelkette (Herstellung, Transport, Lagerung) verstärken.

## Auswirkungen auf Gesundheit und Gesundheitsversorgung

Die Auswirkungen der klimawandelbedingten Veränderungen unserer Umwelt- und Lebensbedingungen in Deutschland auf die individuelle Gesundheit und die Gesundheitsversorgung allgemein sollen hier im Überblick dargestellt werden (siehe auch Infobox 2).

### Hitzeassoziierte Krankheiten und Mortalität

Gesundheitliche Folgen von Hitze sind gut belegt. Erhöhte Temperaturen und Hitzewellen können hitzebedingte Erkrankungen wie Hitzschlag und Hitzekollaps oder Störungen wie Dehydrierung und Hitzeerschöpfung auslösen [12]. Sie können auch vorbestehende hitzesensitive Erkrankungen verschlimmern. Patientinnen und Patienten mit kardiometabolischen Erkrankungen sind besonders gefährdet. Während Hitzewellen kann es zu einer Erhöhung von Blutdruck und Herzfrequenz kommen, in deren Folge das Risiko für Akutereignisse wie Herzinfarkt oder Schlaganfall steigt [25]. Menschen mit Diabetes weisen während Hitzephasen eine verstärkte Neigung zur Bildung von Blutgerinnseln auf. Weiterhin besteht für Menschen mit respiratorischen Erkrankungen eine erhöhte Gefährdung durch Hitze, die z. B. durch pulmonalen Stress oder durch einen eingeschränkten Wärmetransport bei Vorschädigungen der Lunge bedingt wird. Zudem ist bekannt, dass sich Medikamenteninteraktionen und Nebenwirkungen von Arzneistoffen in Hitzeperioden verstärken können [26].

Hohe Lufttemperatur und Luftschadstoffe wirken zusammen [27]. An heißen Tagen besteht häufig eine geringe Luftzirkulation, sodass insbesondere in Städten Luftschadstoffe in höherer Konzentration in der Luft verbleiben und die Feinstaubbelastung steigen kann. Hohe Lufttemperaturen in Kombination mit intensiver Sonneneinstrahlung begünstigen zudem die Steigerung der bodennahen Ozonkonzentration.

Das Ausmaß hitzebedingter Mortalität in Deutschland wurde auf regionaler Ebene und seit 2019 wiederholt auf Bundesebene untersucht [12, 28–31]. In den Hitzesommern 1994 und 2003 wurden für Deutschland insgesamt jeweils rund 10.000 hitzebedingte Sterbefälle geschätzt. Eine Häufung von Sommern mit einer signifikanten Anzahl hitzebedingter Sterbefälle wird seit 2018 angenommen (Todesfälle 2018: 8300, 2019: 6900, 2020: 3600, 2022: 4500).

Auswirkungen von Hitzeperioden zeigen sich auch in einer erhöhten Inanspruchnahme von Gesundheitsleistungen. So wurde anhand von Daten der Gesetzlichen Krankenversicherung der Effekt eines einzelnen weiteren Hitzetages mit einer Höchsttemperatur von mindestens 30 °C auf rund 40 zusätzliche Krankenhausaufnahmen pro 1 Mio. AOK-Versicherte über 65 Jahren geschätzt [32]. Für die 1 % der Versicherten mit dem höchsten Risiko für hitzebedingte Krankenhausaufnahmen lag dieser Wert bei 553. Siehe auch [33].

Hitzebelastungen treten innerhalb Deutschlands je nach geografischer Lage, Topografie und Landnutzung sehr unterschiedlich auf. Hitzewellen werden besonders in südwestlichen und östlichen Regionen Deutschlands beobachtet [13]. Hitzeassoziierte Morbidität, Mortalität und Leistungsinanspruchnahme sind regional sehr unterschiedlich ausgeprägt. Ursächlich sind hierfür nicht allein die Hitzeexposition und die geografischen Merkmale, sondern auch der Anteil der vulnerablen Personen in der jeweiligen Bevölkerung. Als vulnerabel für hitzebedingte Morbidität und Mortalität gelten Vorerkrankte mit Herz-Kreislauf‑, Atemwegs‑, Nieren- und psychischen Erkrankungen sowie Übergewichtige und Diabetiker [12]. Ältere, Pflegebedürftige, Schwangere, Säuglinge, Kleinkinder, Menschen mit körperlichen und geistigen Beeinträchtigungen, Personen mit körperlicher Aktivität im Freien und sozial benachteiligte Personen sind ebenfalls stärker gefährdet [34, 35]. Ursächlich können hier eine verminderte Fähigkeit zur Regulierung der Körpertemperatur, Einschränkungen bei Selbstversorgung und Selbstschutz und die erhöhte Exposition sein.

Die Analyse von versorgungsnahen Gesundheitsdaten kann helfen, gefährdete Bevölkerungsgruppen und Regionen zu identifizieren, die von gesundheitlichen Auswirkungen extremer Hitze besonders betroffen sind.

### Infektionskrankheiten

Die Zunahme von Infektionskrankheiten als Folge des Klimawandels zeigt sich in Form von Vektor- und Nagetier-assoziierten Erkrankungen. Deren Auftreten wird durch die erstmalige oder vermehrte Verbreitung von Erregern durch die Wirte begünstigt. Als Beispiel sei die Inzidenzzunahme von FSME-Erkrankungen angeführt. In Baden-Württemberg und Bayern, den in Deutschland am stärksten betroffenen Bundesländern, stieg zwischen 2001 und 2018 die Inzidenz von FSME altersbereinigt jährlich um 2 % an (95 %-Konfidenzintervall 0,8–3,6 %) [24]. Weiterhin können wasserbürtige Infektionen und lebensmittelassoziierte Infektionen häufiger auftreten.

Die Ausbreitung von Infektionskrankheiten kann bei meldepflichtigen Infektionen anhand der Daten der Gesundheitsämter analysiert werden. Für nicht meldepflichtige Krankheiten können teilweise versorgungsnahe Daten wie Routinedaten der Krankenkassen herangezogen werden, sofern die Erkrankungen zu Behandlungen im Gesundheitssystem geführt haben und deren Dokumentation ausreichend detailliert erfolgt ist (Diagnosekodierung im ICD-Katalog, räumliche und zeitliche Detaillierung).

### Weitere nichtübertragbare Erkrankungen

Neben den bereits erwähnten hitzeassoziierten Erkrankungen hat der Klimawandel Auswirkungen auf weitere nichtübertragbare Erkrankungen in Form von häufiger auftretenden Allergien [4, 7], UV-bedingten bzw. UV-sensitiven Erkrankungen wie Hautkrebs und entzündlichen Hauterkrankungen [5, 23]. Auch Verletzungen und Todesfälle durch Extremwetterereignisse wie Starkregen, Fluten und Waldbrände [20] sowie Atemwegs- und Herz-Kreislauf-Erkrankungen im Zusammenhang mit Luftschadstoffexposition bzw. Interaktionen von erhöhter Lufttemperatur und Luftschadstoffen [8, 27] sind zu beobachten.

Auswirkungen des Klimawandels auf die psychische Gesundheit in Deutschland wurden bislang selten untersucht. In einzelnen Regionen wurden vermehrt aggressive Zwischenfälle, Suizidversuche und Suizide im Zusammenhang mit steigenden Temperaturen berichtet [11]. Sorgen um ihre eigene Gesundheit in Bezug auf Hitzeperioden äußerten 52 % der Befragten in einer 2020 deutschlandweit durchgeführten, repräsentativen Bevölkerungsbefragung von 3000 Personen [36]. Klinische Symptome bei Gedanken an den Klimawandel werden kaum angegeben.

## Datengrundlagen zur Analyse gesundheitlicher Auswirkungen des Klimawandels

Zur Analyse von Auswirkungen des Klimawandels, konkret der veränderten Umwelt- und Lebensbedingungen, auf die Gesundheit und die Gesundheitsversorgung werden Expositionsdaten, Daten zur Gesundheitsversorgung, die potenzielle Effekte abbilden, und weitere diese Effekte modulierende Daten benötigt (siehe Tab. 1).

**Table 1:** 

Expositionsdaten	Wetterdaten (Wolkenbedeckung, relative Feuchtigkeit, Windgeschwindigkeit, Niederschlag, Oberflächendruck)
	Daten zu Extremereignissen (Sturm, Starkregen, Überschwemmungen)
	Temperaturdaten (Tagesmittelwerte, Tagesminima und Tagesmaxima, Anzahl von Hitzetagen)
	Luftschadstoffe
	UV-Index
	Feinstaub
	Pollendaten
	Daten zur Wasserqualität
Versorgungsnahe Daten	Daten zu Morbidität, Mortalität
	Daten zur Inanspruchnahme von Gesundheitsleistungen
	Daten zur Arbeitsunfähigkeit
	Daten zu Versorgungskosten
	*Unterscheidung zwischen:*
	Primärdaten
	Registerdaten und amtlichen Statistiken (z. B. Sterbefallzahlen)
	Sekundärdaten
Modulierende Daten	Sozioökonomische Daten
	Siedlungsstrukturelle Daten
	Versorgungsdichte
	Geländetopografie
	Präventive Maßnahmen (z. B. Hitzeaktionsplan)

Die Datengrundlagen müssen auf räumlicher, zeitlicher und gegebenenfalls individueller Ebene miteinander verschnitten werden, um die Exposition mit möglichen gesundheitlichen Effekten in Beziehung zu setzen. So kann beispielsweise der Effekt einer räumlich und zeitlich begrenzten Hitzewelle auf die kardiale Mortalität in der betroffenen Region unter Berücksichtigung städtebaulicher Gegebenheiten untersucht werden.

Im Weiteren werden dem Beitragsschwerpunkt folgend versorgungsnahe Daten betrachtet.

### Versorgungsnahe Daten

Ziel der Versorgungsforschung ist es meist, die Gesundheitsversorgung unter Alltagsbedingungen zu analysieren. Dabei stützt sich die Versorgungsforschung auf eine breite Palette von Datenquellen, die die Versorgungspraxis möglichst unmittelbar abbilden. Als Oberbegriff für Datenbestände, die außerhalb experimenteller Studien im Rahmen bestehender Versorgungsabläufe erhoben werden, wurde der Terminus „versorgungsnahe Daten“ eingeführt [37]. Bei den versorgungsnahen Daten werden Primärdaten, Registerdaten und Sekundärdaten unterschieden [38]. Primärdaten sind Daten, die erst im Rahmen von Forschungsvorhaben erhoben werden (z. B. Befragung zur Lebensqualität). Registerdaten sind Daten existierender medizinischer, krankheitsbezogener und anderer Register (z. B. klinische Krebsregister). Sekundärdaten sind Daten, die im Rahmen von administrativen Prozessen entstehen und für die Forschung sekundär genutzt werden (z. B. Abrechnungsdaten zu Gesundheitsleistungen). Eine sekundäre Datennutzung liegt vor, wenn die Daten nicht entsprechend dem originären Erhebungszweck, sondern für andere Zwecke wie zum Beispiel die wissenschaftliche Forschung verwendet werden. Bei der Versorgungsforschung mit Abrechnungsdaten findet eine solche sekundäre Datennutzung statt.

Eine Übersicht über die medizinischen Register in Deutschland bietet die im Auftrag des Bundesministeriums für Gesundheit entwickelte Registerdatenbank der medizinischen Register in Deutschland [39]. Sie listet im September 2023 insgesamt 410 Einträge. Die Register unterscheiden sich gemäß ihren Zielsetzungen hinsichtlich der betrachteten Populationen, der Dateninhalte und der regionalen und zeitlichen Abdeckung deutlich. Eine Zusammenführung verschiedener Register findet am Zentrum für Krebsregisterdaten am Robert Koch-Institut für die Daten der Landeskrebsregister statt.

Routinedaten der Krankenversicherungen beinhalten Daten der Abrechnung von Gesundheitsleistungen und damit im Zusammenhang stehender Leistungen (z. B. Krankentransporte), Angaben zur Arbeitsunfähigkeit und Stammdaten der Versicherten (z. B. Todesdatum). Sie bilden das versichertenbezogene Versorgungsgeschehen innerhalb eines Leistungssektors und zwischen den Leistungssektoren für alle beteiligten Leistungserbringer nahezu lückenlos ab und stellen eine Vollerhebung der zu Lasten der Krankenversicherungen abgerechneten Leistungen aller krankenversicherten Personen dar [40]. Zu den erfassten Dateninhalten gehören Diagnosen und Therapiedaten, Verordnungsdaten (Arznei‑, Heil- und Hilfsmittel), die Art der Leistungserbringung (z. B. Notfall, Rettungsdienst), mit Einschränkungen Ort und Zeitpunkt der Leistungserbringung, mit der Krankenversicherung abgerechnete Kosten, Arbeitsunfähigkeitstage sowie der Wohnort der Versicherten.

Diese Routinedaten eignen sich prinzipiell für Longitudinalanalysen und für raumbezogene Analysen. Sie erlauben Auswertungen zur Mortalität, zur Prävalenz und Inzidenz von Erkrankungen, zur Inanspruchnahme von Gesundheitsleistungen und zu Gesundheitsausgaben. Aufgrund der Erfassung der kompletten Versichertenpopulationen ist die Betrachtung von Personengruppen möglich, die in Primärdatenerhebungen selten oder nur schwer erfasst werden können (z. B. Hochaltrige, Personen mit kognitiven Einschränkungen, Personen im Pflegeheim, Personen mit seltenen Erkrankungen).

Die Analyse von Routinedaten der Krankenversicherungen unterliegt andererseits erheblichen Limitationen [41]. Vor dem Hintergrund des Erhebungskontextes sind die Validität und die Datenqualität zu hinterfragen. Beispielsweise kann bei pauschalierter Abrechnung ambulanter ärztlicher Leistungen nicht immer ein taggenauer Leistungsbezug hergestellt werden. Ambulante Diagnosen liegen grundsätzlich nur auf Quartalsebene vor. Leitlinien und Empfehlungen zur Planung und Durchführung von Sekundärdatenanalysen und ihrer Publikation sind darum integraler Bestandteil der Versorgungsforschung mit Routinedaten [42–44].

Routinedaten der gesetzlichen Krankenversicherung (GKV) werden gemäß §§ 303 a–f SGB V im Forschungsdatenzentrum Gesundheit (FDZ Gesundheit) am Bundesinstitut für Arzneimittel und Medizinprodukte (BfArM) zusammengeführt [45]. Auf diesem Weg sollen pseudonymisierte Gesundheitsdaten von 73 Mio. gesetzlich Versicherten zu Forschungszwecken zur Verfügung gestellt werden. Zudem sollen im FDZ Gesundheit auch Daten aus der elektronischen Patientenakte der Versicherten ergänzt werden.

Durch die Kombination von versorgungsnahen Daten verschiedener Quellen können Limitationen einzelner Datenbestände überwunden werden. Ein Beispiel hierfür stellt das Datenlinkage zwischen GKV-Routinedaten und Krebsregisterdaten dar, wodurch die Verlaufs- und Inanspruchnahmedaten aus der Krankenversicherung mit den in den GKV-Daten nicht enthaltenen Informationen zur Histologie und zum Stadium der Tumorerkrankung aus den Krebsregistern verknüpft werden können. Der Gesetzgeber plant die Durchführung eines solchen Datenlinkage ab 2024 wesentlich zu vereinfachen [38]. Weiterhin sollen die Daten der gesetzlichen Pflegeversicherung in das FDZ Gesundheit integriert werden.

## Versorgungsforschung zum Klimawandel mit Sekundärdaten

**Figure 1:**
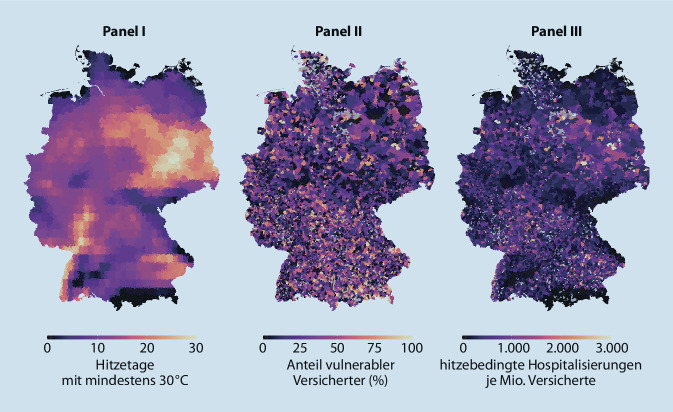

Die Analyse gesundheitlicher Auswirkungen von Umwelt- und Lebensbedingungen ist Bestandteil der Versorgungsforschung. Beispiele hierfür sind Sekundärdatenstudien zum Effekt von Fluglärm als Risikofaktor für Herzinsuffizienz und Hypertonie [46] oder zum Einfluss veränderter Luftschadstoffbelastung durch die Einführung von Umweltzonen auf Ausgaben für Arzneimittel gegen Herz- und Atemwegserkrankungen [47]. Klauber und Koch analysierten auf der Grundlage von Sekundärdaten von AOK-Versicherten, wie unterschiedlich sich Temperaturen von mindestens 30 °C auf die Hospitalisierungsrate der über 65-Jährigen auswirken [32]. Weiteres Ziel der Studie war es, individuelle und regionale Risikofaktoren für hitzebedingte Gesundheitsschäden bei der älteren Bevölkerung zu identifizieren (Abb. 1).

Die Abbildung zeigt vereinfacht beispielhaft, wie der Einfluss einer Exposition (hier Hitzetage mit mindestens 30 °C) unter Berücksichtigung eines modulierenden Faktors (hier Anteil hitzevulnerabler Versicherter) auf den in versorgungsnahen Daten operationalisierten Endpunkt (hier Hospitalisierung) wirkt, woraus die stationäre „Überinanspruchnahme“ abgeschätzt wird. Die Abrechnungsdaten wurden über Klinikstandort und Aufnahmetag mit ERA5^1^-Wetterdaten verschnitten.

Die Datengrundlagen und Methoden der Versorgungsforschung scheinen besonders geeignet, die verschiedenen Auswirkungen des Klimawandels auf die Gesundheitsversorgung zu untersuchen, für die breiter Forschungsbedarf festgestellt wird [11, 20, 31, 48] (siehe Tab. 2).

**Table 2:** 

Aspekte des Klimawandels	Untersuchungsgegenstand
*Hitzebelastung*	(Möglichst kleinräumige) Effekte auf Mortalität, verlorene Lebensjahre, Morbidität Effekte auf Leistungsinanspruchnahme, Arbeitsunfähigkeit, direkte und indirekte Kosten
	Latenz hitzeassoziierter Erkrankungen und Behandlungsereignisse in Bezug auf Beginn und Ende einer Hitzewelle
	Langzeiteffekte von hohen Temperaturen und Hitze
	Einfluss der Tageshöchsttemperatur, Dauer von Hitzewellen, Tropennächte
	Einfluss des saisonalen zeitlichen Auftretens von Hitzewellen
	Identifikation vulnerabler Personengruppen
	Rolle von Vorerkrankungen (z. B. Herzinsuffizienz, Herzinfarkte, Schlaganfälle, Diabetes, Nierenerkrankungen, COPD, Asthma)
	Betroffenheit von Schwangeren
	Betroffenheit von betreuten Personen in Gesundheits- und Pflegeeinrichtungen
	Betroffenheit von Mitarbeitenden in Gesundheits- und Pflegeeinrichtungen (Gewährleistung der medizinischen Versorgung und der Pflege während Hitzeperioden)
	Betroffenheit der städtischen Bevölkerung
	Auswirkung zusätzlicher Umweltfaktoren (z. B. Luftschadstoffbelastung)
	Interaktionen bei Einnahme verschiedener hitzesensitiver Medikamente
*Allergien*	Monitoring des Auftretens allergischer Reaktionen in Abhängigkeit von der Exposition gegenüber neuen oder sich ausbreitenden Allergenquellen
	Effekte sich verändernder Pollensaisons (Beginn, Dauer) auf das Auftreten allergischer Reaktionen
	Einfluss von Lufttemperatur und Luftschadstoffen auf das Auftreten von Pollenallergiesymptomen
*UV-Strahlenbelastung*	Monitoring UV-Strahlungs-assoziierter Erkrankungen
	Einfluss wetterabhängiger Verhaltensgewohnheiten auf die tatsächliche UV-Strahlungsbelastung
	Einfluss klimawandelbedingter Veränderungen des Wetters auf Verhaltensgewohnheiten
*Mikroorganismen und Vektoren*	Systematisches Monitoring vektorübertragbarer Infektionen
	Monitoring insbesondere bzgl. klimawandelbedingt neu oder stärker verbreiteter Infektionskrankheiten
*Psychische Belastungen*	Effekte durch Klimawandel bedingter Extremereignisse auf die psychische Gesundheit (z. B. posttraumatische Belastungsstörungen, Depression)
*Maßnahmen zur Bewältigung gesundheitlicher Auswirkungen* *Störungen des Gesundheitssystems*	Effekte von Präventionsmaßnahmen (Verhaltens- und Verhältnisprävention)
	Effekte von Hitzeaktionsplänen in der Vermeidung gesundheitlicher Gefährdungen
*Störungen des Gesundheitssystems*	Effekte von Extremereignissen auf die Leistungsfähigkeit des Gesundheitssystems (Großschadensereignisse, Überlastung)

Die Gesundheitssysteme müssen sich an die sich verändernden Muster der klimabedingten Gesundheitsauswirkungen anpassen. Anpassungsbedarf besteht auf übergeordneter Ebene bei der Organisation der Gesundheitsversorgung und der Vorhaltung von Infrastrukturen. Datengestützte Analysen können in die Planung von Behandlungs- und Rettungskapazitäten einfließen und sicherstellen, dass Krankenhäuser und Einrichtungen des Gesundheitswesens auf ein erhöhtes Patientenaufkommen bei klimabedingten Gesundheitsnotfällen vorbereitet sind. Prognosemodelle für Effekte von Hitzewellen und anderen klimabedingten Gesundheitsgefährdungen auf kurzfristige Änderungen der Leistungsinanspruchnahme könnten zur Entwicklung von Frühwarnsystemen genutzt werden, um eine rechtzeitige Ressourcenzuweisung zu ermöglichen. Die Resilienz der Gesundheitsinfrastruktur könnte durch die Vermeidung kurz- und langfristiger Überlastungen gestärkt werden.

Erkenntnisse über besonders gefährdete Personengruppen und Risikofaktoren können in der Gesundheitsförderung als Grundlage für Kampagnen und Aufklärungsmaßnahmen dienen, die auf die Verringerung klimabedingter Gesundheitsrisiken abzielen und dazu motivieren, Vorsorgemaßnahmen zu ergreifen. Die Gesundheits- und Pflegeeinrichtungen können gezielte Maßnahmen ableiten (z. B. Vorbeugung von Dehydrierung) [34]. Langfristige Anpassungsstrategien wie die Entwicklung nachhaltigerer Städte, die besser auf die steigenden Temperaturen und Extremereignisse vorbereitet sind, könnten gezielter verfolgt werden [49].

## Fazit

Die Analyse von versorgungsnahen Daten des Gesundheitswesens liefert wertvolle Erkenntnisse über die Auswirkungen des Klimawandels auf die Gesundheit und die Gesundheitsversorgung. Forschungsthemen wie hitzeassoziierte Krankheiten, durch Vektoren übertragene Krankheiten, Effekte von Extremereignissen sowie der Forschungsbedarf bezüglich gefährdeter Bevölkerungsgruppen verdeutlichen die Vielschichtigkeit der potenziellen Belastungen des Gesundheitswesens. Da der Klimawandel bereits heute – und zukünftig noch stärker – zu gesundheitlichen Gefahren für die Bevölkerung führt, wird datengestützte Forschung eine zentrale Rolle beim Verständnis, der Bewältigung und der Anpassung an seine gesundheitlichen Folgen spielen. Politische Entscheidungsträger und Gesundheitsdienstleister müssen auf der Grundlage dieser Erkenntnisse handeln, um das Wohlergehen der Bevölkerung in einem sich verändernden Klima zu sichern.

### Infobox 1 Auswirkungen des Klimawandels auf die Umweltbedingungen in Deutschland

- Zunahme von Hitzewellen (in Bezug auf Anzahl, Dauer, Temperatur)
- Zunahme von Trockenheit und Dürren
- Zunahme von Waldbränden
- Abnahme von Schnee- und Eistagen
- Zunahme von Starkregen
- Verschlechterung der Wasser- und Luftqualität
- Einfluss auf UV-Strahlung
- Zunehmende Verbreitung von Allergenen
- Zunehmende Verbreitung von Vektoren (z. B. Stechmücken, Zecken)
- Zunehmende Verbreitung von lebensmittelassoziierten Bakterien

### Infobox 2 Auswirkungen des Klimawandels auf die Gesundheitsversorgung in Deutschland

- Zunahme von hitzeassoziierten Erkrankungen und Todesfällen
- Zunahme von Medikamenteninteraktionen und Nebenwirkungen von Arzneimitteln
- Zunahme von Infektionserkrankungen
- Zunahme von allergischen Erkrankungen
- Zunahme von Hautkrebs und Augenerkrankungen aufgrund von UV-Strahlen
- Verletzungen und Todesfälle aufgrund von Extremwetterereignissen
- Zunahme von Luftschadstoff-assoziierten Erkrankungen
- Einfluss auf psychische Belastungen
- Störungen des Gesundheitssystems (z. B. Überlastung bei Großschäden)

## Footnotes

1 ERA5: European Centre for Medium-Range Weather Forecasts (ECMWF) Reanalysis 5.
